# Association between methadone dose and concomitant cocaine use in methadone maintenance treatment: a register-based study

**DOI:** 10.1186/1747-597X-9-46

**Published:** 2014-12-04

**Authors:** Marcus Baumeister, Marc Vogel, Kenneth M Dürsteler-MacFarland, Urs Gerhard, Johannes Strasser, Marc Walter, Gerhard A Wiesbeck, Sylvie A Petitjean

**Affiliations:** Outpatient Addiction Treatment Center Reinach, Psychiatry Baselland, Baselstrasse 1, 4153 Reinach, Switzerland; Division of Substance Use Disorders, Psychiatric Hospital of the University of Basel, Wilhelm Klein-Strasse 27, 4012 Basel, Switzerland; Department of Psychology, University of Basel, Missionsstrasse 60/62, 4055 Basel, Switzerland

**Keywords:** Cocaine use, Methadone dose, Methadone maintenance treatment, Opioid dependence

## Abstract

**Background:**

Concomitant cocaine use is a major problem in clinical practice in methadone maintenance treatment (MMT) and may interfere with successful treatment. Data from European methadone populations is sparse. This register-based study sought to explore the association between prescribed methadone dose and concomitant cocaine and heroin use in the methadone population of Basel City.

**Methods:**

The study included 613 methadone patients between April 1, 2003 and March 31, 2004. Anonymized data was taken from the methadone register of Basel City. For analysis of the prescribed methadone dose distribution, the patient sample was split into three methadone dosage groups: a low dose group (LDG) (n = 200; < 60 mg/day), a medium dose group (MDG) (n = 273; 60 to 100 mg/day), and a high dose group (HDG) (n = 140; > 100 mg/day). Concomitant drug use was based on self-report.

**Results:**

Analysis showed a significant difference in self-reported cocaine use between groups (p < 0.001). Patients in the LDG reported significantly fewer cocaine consumption days compared to the MDG (p < 0.001) and the HDG (p < 0.05). Patients in the HDG reported significantly fewer heroin consumption days than those in the LDG (p < 0.01) and the MDG (p < 0.001). In logistic regression analysis, cocaine use was significantly associated with heroin use (OR 4.9).

**Conclusions:**

Cocaine use in methadone patients may be associated with heroin use, which indicates the importance of prescribing appropriate methadone dosages in order to indirectly reduce cocaine use.

## Introduction

Opioid dependence is a serious chronic illness with a multitude of somatic and psychosocial risks and which in most cases requires long-term treatment [1–4]. Methadone maintenance treatment (MMT) is an effective treatment for opioid dependent patients, particularly when given with other psychosocial services [1–7]. MMT has proven beneficial in reducing illicit opioid use and associated harms, and is considered to be the first line treatment [2, 3, 8, 9] Nevertheless, some methadone patients continue to abuse heroin and other non-opiate drugs, such as cocaine, during treatment.

In Switzerland, the prevalence of opioid dependence was 0.9% in 2012 (men 1.6%, women 0.3%) [10] and cocaine has been used by approximately 4.1% of citizens aged between 15 and 64 years at least once in their lives [11]. Prescription of methadone for opioid dependence has been regulated by federal and state laws since 1975. Approximately 17,000 patients in Switzerland are on opioid substitution treatment; this number has been nearly constant since 2000. Several medications are used for maintenance treatment in Switzerland, including full μ-receptor agonists (e.g. oral methadone and slow release morphine, oral and intravenous diacetylmorphine), and the partial μ-receptor agonist and κ-receptor antagonist, buprenorphine [12]. Most patients receive methadone (90%). MMTs are covered by health insurance and are provided by general practitioners (GPs) and specialized centers [6]. Inclusion criteria for entering a MMT are opioid dependence, being at least 18 years of age, a positive toxicology test for heroin use, and failure in previous abstinence-oriented treatments. The prescribed and administered methadone is a 2% racemic methadone hydrochloride solution. In clinical practice, individualised treatment plans are developed and methadone doses are prescribed on the basis of clinical impression, according to Swiss recommendations [13]. Basel City has 200,000 inhabitants, with approximately 2000 heroin users. Most patients are treated in specialized centers and one third in an office-based setting. In Basel City, the average prescribed methadone dose has increased over the past two decades from 59 ± 31 mg/day [14] to 74 ± 47 mg/day [6].

The tendency toward higher methadone doses has been supported by randomised clinical trials [15–18] and observational studies [19–26]. While MMT has been demonstrated to be an effective treatment for opioid dependence, its impact on the treatment outcome of cocaine abuse is not as clear. There is some evidence that patients abusing cocaine during MMT are more likely to drop out [27, 28] and present a higher risk profile regarding HIV and psychological disturbances [29], as well as higher heroin use [20]. A positive effect of MMT on concomitant cocaine use was observed in 5 observational studies [30–35], 1 meta-analysis [17] and 1 RCT [36]. On the other hand, a mild effect was found in 2 observational studies [37, 38] and no effect in 2 observational studies [32, 39] and 2 RCTs [40, 41]. There have been few studies on the influence of different methadone dose levels and its association with concurrent cocaine use. Castells et al. [42] found no effect on cocaine abstinence at methadone doses > 50 mg/d, as did Kennedy et al. [41] in an RCT at methadone doses > 100 mg/d. Nevertheless, Peles et al. [36] found an effect on cocaine use in MMT at methadone doses > 100 mg/d. Data from European methadone populations is sparse.

This register-based study sought to explore the association between different prescribed methadone dose levels and concomitant cocaine and heroin use in the methadone population of Basel City between April 1, 2003 and March 31, 2004. We were especially interested in the effect of higher methadone doses on concomitant cocaine use, because not enough is known on this topic. We hypothesised that patients with prescribed methadone doses > 100 mg/day would have better treatment outcomes. To answer these research questions, we split the sample into three subsamples. Methadone dosage groups were defined according to a National Institutes of Health (NIH) expert panel that has stated that a daily methadone dose of at least 60 mg is best practice in methadone maintenance [43]. The dose groups were a low dose group (LDG) (10 mg/day to 59 mg/day), a medium dose group (MDG) (60 to 100 mg/day), and a high dose group (HDG) (101 mg/day to 260 mg/day).

## Methods

### Subjects

All data was taken from the methadone register of the health authorities of Basel City, which was discontinued in 2004. Data had been collected since 1995. The data collection and evaluation are in accordance with the data protection law of the Canton of Basel City and were approved by the local ethics committee “Ethikkommission beider Basel EKBB” Hebelstrasse 53 CH-4056 Basel Switzerland http://www.ekbb.ch (ethical review committee of both Basel (Basel-City and Basel-Countryside)). As stipulated by legislation, prescribing treatment providers in Basel City are required to submit a registration form to the health authorities each time a heroin-dependent patient begins and ends MMT. The form collects information about methadone patients and their treatment. For monitoring purposes, methadone prescribers were further invited until 2004 to provide anonymized patient and treatment data to the register every 12 months by means of a 2-page questionnaire. Methadone prescribers were instructed to carry out the interviews within one month and send them back to the health authorities. This structured questionnaire contained a core of questions about gender, nationality, marital status, educational level, work situation, age at first heroin and cocaine use, treatment facility [specialized centers, office based treatment], year entering MMT, currently prescribed methadone dose (mg/d), prescribed psychiatric comedication, allowed take-home days per week (0–7 days), number of consultations in the past 6 months, and self-reported substance use (heroin, cocaine, alcohol, cannabis) during the previous 30 days (according to the European Addiction Severity index [44]). The present evaluation includes data of MMT patients from April 1, 2003 to March 31, 2004. Throughout that period, three clinics (two public, one private) and 81 office-based practitioners conducted MMT in Basel City.

### Procedures and statistical analyses

Anonymised data from prescription for opioids were collected and analysed by the Addiction Research Center of the Hospital of the University of Basel. A total of 974 patients were surveyed. The response rate was 91% (n = 886). Overall 273 out of 886 patients had to be excluded from the statistical analyses by the study investigators due to missing variables (e.g. gender, age [n = 12], daily prescribed methadone dosage [n = 17], self-reported illicit drug use in the past 30 days [n = 78], and length of stay in treatment [n = 79]). A further 68 patients were excluded from the analyses, because they had been given a prescription for opioid substitution treatment other than with methadone (buprenorphine, n = 45; morphine, n = 23), and 19 patients were excluded because they were tapering methadone to end MMT. The final sample included 613 patients and was split into three methadone dose groups (LDG, MDG and HDG).

Dependent variables were concomitant cocaine use (consumption days) and concomitant heroin use (consumption days). Continuous (interval-scaled) data were analyzed by one way analysis of variance, and post-hoc tests (Duncan Scheffé’s); categorical data were analyzed by χ^2^ statistics and non-parametric tests (Kruskal-Wallis test, Mann–Whitney test).

To model the determinants of cocaine use in MMT, we used a backward fitting procedure that applied likelihood ratio tests to develop a binary logistic regression model, with cocaine use (coded as a dichotomous variable) as the dependent variable. Initially we included the following predictor variables, that were either associated in bivariate analysis or known from literature: age (years); gender (female); employment status (yes = 1); office-based settings (yes = 1); days with take-home (days); prescribed methadone dose group (low methadone dose as the reference category); and concomitant heroin use (no = 0). Because of a large number of missing values, we refrained from including the variables route of administration, age at first heroin and cocaine use. Data analyses were conducted using SPSS (version 17, SPSS Inc, Chicago, IL). The level of significance was set at *p <* 0.05.

## Results

### Socio-demographic characteristics

Of 613 methadone patients in MMT, 32.6% were in the LDG (n = 200), 44.5% in the MDG (n = 273), and 22.8% in the HDG (n = 140). Patients were aged from 22 to 61 years (38.9 ± 6.6); two thirds were men (67.9%); 10.4% were married; the mean years of education were 10.8 ± 1.6, and 20.6% were employed (see Table 1). Overall, demographic characteristics did not differ between the three methadone dosage groups, except for the variables employment status and age at first heroin use, with a significantly higher proportion of patients in the LDG than in the MDG and HDG who were employed or who had started using heroin significantly later in their lives (see Table 1).

**Table 1:** **Sociodemographic characteristics** ^**a**^

Variables	Low dose group (LDG) (n = 200)	Medium dose group (MDG) (n = 273)	High dose group (HDG) (n = 140)	Total sample (N = 613)	***p*** value
Gender, n (%) male	132 (66.0)	188 (68.9)	96 (68.6)	416 (67.9)	p > 0.05
Age, mean (SD), y	39.4 (±6.9)	38.4 (±6.4)	38.9 (±6.4)	38.9 (±6.6)	p > 0.05
Nationality, n (%) Swiss	169 (86.2)	230 (86.5)	118 (84.9)	517 (86.0)	p > 0.05
Marital status, n (%) Married	24 (12.0)	28 (10.3)	12 (8.6)	64 (10.4)	p > 0.05
Education, mean (SD), y	10.9 (±1.5)	10.9 (±1.6)	10.6 (±1.6)	10.8 (±1.6)	p > 0.05
Employed, n (%)^b^	54 (29.0)	49 (18.8)	17 (12.6)	120 (20.6)	p < 0.001
Age at first heroin use, mean (SD), y^c^	20.8 (±5.3)	19.2 (±4.3)	18.1 (±4.1)	19.5 (±4.7)	p < 0.01
Age at first cocaine use, mean (SD), y^d^	22.3 (±6.9)	21.3 (±5.7)	19.7 (±4.3)	21.3 (±5.9)	p > 0.05

^a^The groups did not differ in any variable at the p < 0.05 level, except as noted.

^b^Significant difference between patients in the LDG, MDG and HDG (Kruskal-Wallis-Test; χ^2^ = 15.587; df = 2; p < 0.001). The Mann Whitney test revealed that patients in the LDG were significantly more often employed than patients in the MDG (Z = − 2.878; p < 0.01) or in the HDG (Z = −3.603; p < 0.001).

^c^Significant difference between patients in the LDG, MDG and HDG for age at first heroin use (ANOVA; F_(2,277)_ = 6.206; p < 0.01). Post-hoc tests (Duncan Scheffé’s) revealed that patients in the LDG started heroin use significantly later in their lives than patients in the MDG (p < 0.05) or in the HDG (p < 0.01).

^d^No difference between the LDG, MDG and HDG for age at first cocaine use, but a trend for patients in the HDG to start cocaine use earlier in their lives (trend, p = 0.054).

### Clinical characteristics

Two thirds of the patients received their methadone in specialized centers and prescribed mean methadone doses differed significantly between groups (p < 0.001; range: 10 to 260 mg/day; mean: 81.9 ± 46.7 mg/d) (see Table 2). Two thirds of the patients had a prescribed psychiatric comedication, with the HDG presenting the highest proportion. Allowed methadone take-home days per week also varied significantly between groups (p < 0.001). Patients in the HDG had significantly fewer allowed take-home days than patients in the LDG (p < 0.001) but not those in the MDG (p > 0.05). Patients had been in MMT for between 1 and 29 years (10.2 ± 4.7), with significant differences between groups (p < 0.001). Patients in the HDG had a significantly longer period in MMT than the LDG (p < 0.001) but not the MDG (p > 0.05). Patients had had a mean of 6.4 ± 4.7 consultations within the 6 months prior to the interview, with significant differences between groups (p < 0.01). Patients in the HDG had significantly more consultations than patients in the LDG (p < 0.01) and in the MDG (p < 0.05). Patients reported a mean of 9.1 ± 12.1 days of alcohol use in the past 30 days prior to the interview, with significant differences between groups (p < 0.01). Patients in the HDG reported significantly more alcohol consumption days than those in the LDG (p < 0.01), but not than those in the MDG (p > 0.05). Patients reported a mean of 8.3 ± 11.2 cannabis consumption days, with non-significant group differences (see Table 2).

**Table 2:** **Clinical characteristics**

Variables	Low dose group (LDG) (n = 200)	Medium dose group (MDG) (n = 273)	High dose group (HDG) (n = 140)	Total sample (N = 613)	***p*** value
Specialized centers, n (%)^a^	114 (57.0)	189 (69.2)	104 (74.3)	407 (66.4)	p < 0.01
Methadone dose mg/ day, mean (SD)^b^	35.0 (±12.1)	81.3 (±14.8)	150.0 (±33.3)	81.9 (±46.6)	p < 0.001
Prescribed comedication, n (%)^c^	77 (38.7)	164 (60.1)	108 (77.1)	349 (57.0)	p < 0.001
Allowed take-home days per week, mean (SD)^d^	5.8 (±3.7)	5.1 (±3.2)	4.5 (±2.7)	5.2 (±3.3)	p < 0.001
Length of stay in MMT, mean (SD), y^e^	9.3 (±4.5)	10.2 (±4.7)	11.3 (±4.8)	10.2 (±4.7)	p < 0.001
No. of consultations within 6 months, mean (SD)^f^	5.9 (±4.2)	6.2 (±4.2)	7.7 (±6.7)	6.4 (±4.9)	p < 0.01
Alcohol use in the past 30 days, mean (SD)^g^	7.4 (±11.1)	8.9 (±11.1)	11.8 (±13.3)	9.1 (±12.1)	p < 0.01
Cannabis use in the past 30 days, mean (SD)^h^	8.5 (±12.1)	8.9 (±12.0)	11.8 (±13.3)	8.3 (±11.2)	p > 0.05

Significance level p < 0.05.

^a^Significant difference between patients in the LDG, MDG and HDG (Kruskal-Wallis test; χ^2^ = 12.782; df = 2; p < 0.01). The Mann Whitney test revealed that patients in the LDG were significantly more often treated in office-based settings than patients in the MDG (Z = −2.736; p < 0.01) or in the HDG (Z = −3.266; p < 0.001).

^b^Significant difference between patients in the LDG, MDG and HDG for prescribed methadone dose (ANOVA; F_(2,610)_ = 1343.158; p < 0.001).

^c^Significant difference between patients in the LDG, MDG and HDG (Kruskal-Wallis test; χ^2^ = 51.360; df = 2; p < 0.001). The Mann Whitney test revealed that patients in the LDG had a significantly lower proportion of prescribed comedication than patients in the MDG (Z = −4.584; p < 0.001) or in the HDG (Z = −6.990; p < 0.001).

^d^Significant differences between patients in the LDG, MDG and HDG for allowed take-home methadone days per week (ANOVA; F_(2,603)_ = 6.750; p < 0.001). Post-hoc tests (Duncan Scheffé’s) revealed that patients in the HDG had significantly fewer take-home days than patients in the LDG (p < 0.001) but not in the MDG (p > 0.05).

^e^Significant differences between patients in the LDG, MDG and HDG for length of stay in MMT (ANOVA; F_(2,611)_ = 7.734; p < 0.001). Post-hoc tests (Duncan Scheffé’s) revealed that patients in the HDG had a significantly longer length of stay in MMT than patients in the LDG (p < 0.001) but not in the MDG (p > 0.05).

^f^Significant differences between patients in the LDG, MDG and HDG for the number of consultations in the past 6 months (ANOVA; F_(2,603)_ = 5.907; p < 0.01). Post-hoc tests (Duncan Scheffé’s) revealed that patients in the HDG had significantly more consultations than patients in the LDG (p < 0.01) or in the MDG (p < 0.05).

^g^Significant differences between patients in the LDG, MDG and HDG for alcohol use in the past 30 days (ANOVA; F_(2,598)_ = 5.087; p < 0.01). Post-hoc tests (Duncan Scheffé’s) revealed that patients in the HDG had significantly more alcohol consumption days than patients in the LDG (p < 0.01) but not in the MDG (p > 0.05).

^h^No differences between patients in the LDG, MDG and HDG for cannabis use in the past 30 days (ANOVA; F_(2,604)_ = 0.160; p > 0.05).

### Concomitant cocaine use

**Figure 1:**
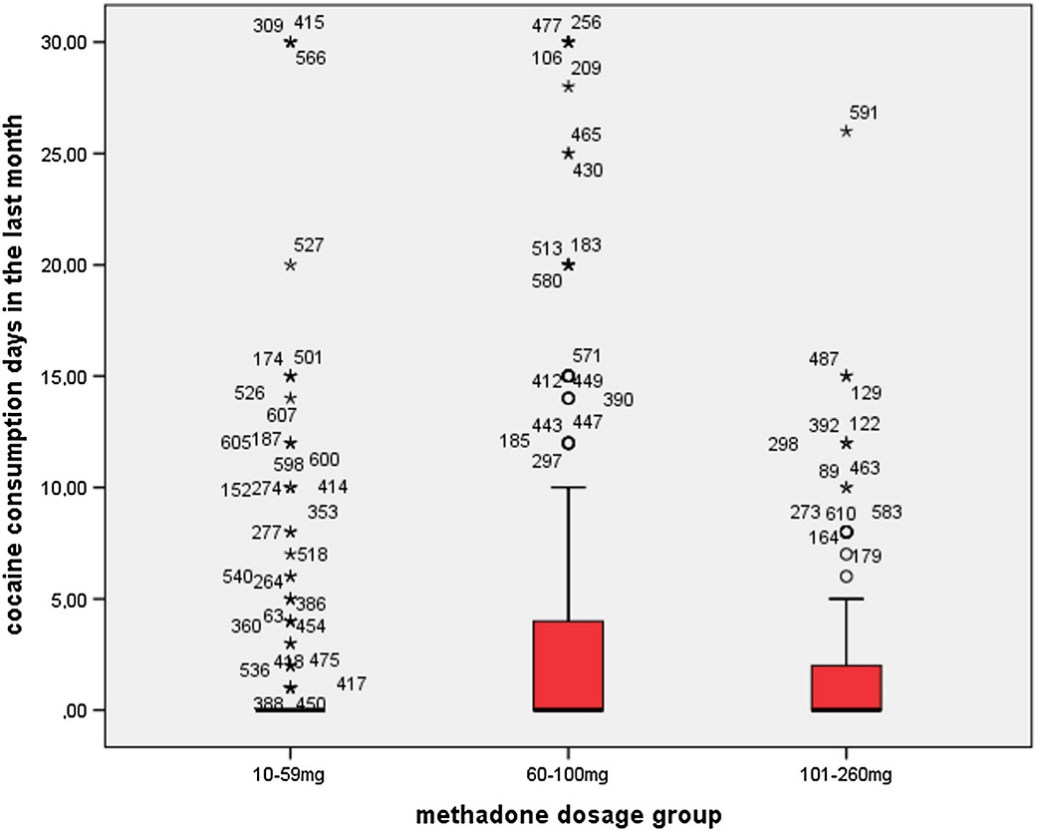
**Self-reported cocaine use in 613 methadone-maintained patients during the 30 days prior to the interview differed significantly with the prescribed methadone dose (mg/ day) (Kruskal-Wallis Test; χ** ^**2**^ **= 15.963; df = 2; p < 0.001).** Patients in the low dose group reported significantly less cocaine use compared to the medium dose group (Mann–Whitney; Z = −3.915; p < 0.001) and the high dose group (Mann–Whitney; Z = −2.058; p < 0.05).

Two thirds of the patients reported no concomitant cocaine use at all in the 30 days prior to the interview (n = 394, 64.3%), and one third (n = 219, 35.7%) reported at least 1 cocaine consumption day. In the LDG, 24.5% (n = 49) reported cocaine use, in the MDG 42.9% (n = 117) and in the HDG 37.9% (n = 53). Patients reported a mean of 2.8 ± 5.9 cocaine consumption days in the past 30 days. As shown in Figure 1, cocaine use differed significantly between methadone dose groups (p < 0.001). Patients in the LDG reported significantly fewer cocaine consumption days than those in the MDG (p < 0.001) and the HDG (p < 0.05). Patients treated in specialized centers exhibited significantly higher proportions of cocaine use (42.0% versus 23.3%; χ^2^ = 20.860; df = 1; p < 0.001), compared to patients in office-based settings.

Table 3 displays the results of binary logistic regression, with cocaine use as dependent variable. The odds for taking cocaine greatly increased with concomitant heroin use (OR 4.9). Furthermore, the odds for cocaine use were increased in patients with prescriptions of both medium and high methadone doses as compared to the LDG, and in those treated in specialized centers. The odds for cocaine use decreased with the number of take-home days. There was a tendency for lower odds in employed patients, but did not reach statistical significance. The other variables were not associated and thus dropped from the model.

**Table 3:** **Odds ratios (OR) and 95% confidence intervals (CI) of binary logistic regression analyses with cocaine use as dependent variable**

		Cocaine use (n = 574, χ^2^ = 2.53, p = 0.96^a^)			
Variable		OR	95% CI		p ^b^
			Lower	Upper	
Concomitant heroin use^c^		4.890	3.245	7.369	<0.0001
Methadone dose^d^	Medium dose (60–100 mg/d)	2.301	1.430	3.701	0.00059
	High dose (>100 mg/d)	2.085	1.190	3.656	0.011
Specialized centers^e^		1.881	1.185	2.986	0.0074
Days with take-home		.822	.754	.897	<0.0001
Employed^f^		.616	.364	1.043	0.071

Significance level set at p < 0.05.

^a^Hosmer-Lemeshow test.

^b^P-value from Wald test with 1 degree of freedom.

^c^No heroin use as reference category.

^d^Low-dose (<60 mg/d) as reference category.

^e^Office-based setting as reference category.

^f^Unemployed/pension as reference category.

### Concomitant heroin use

**Figure 2:**
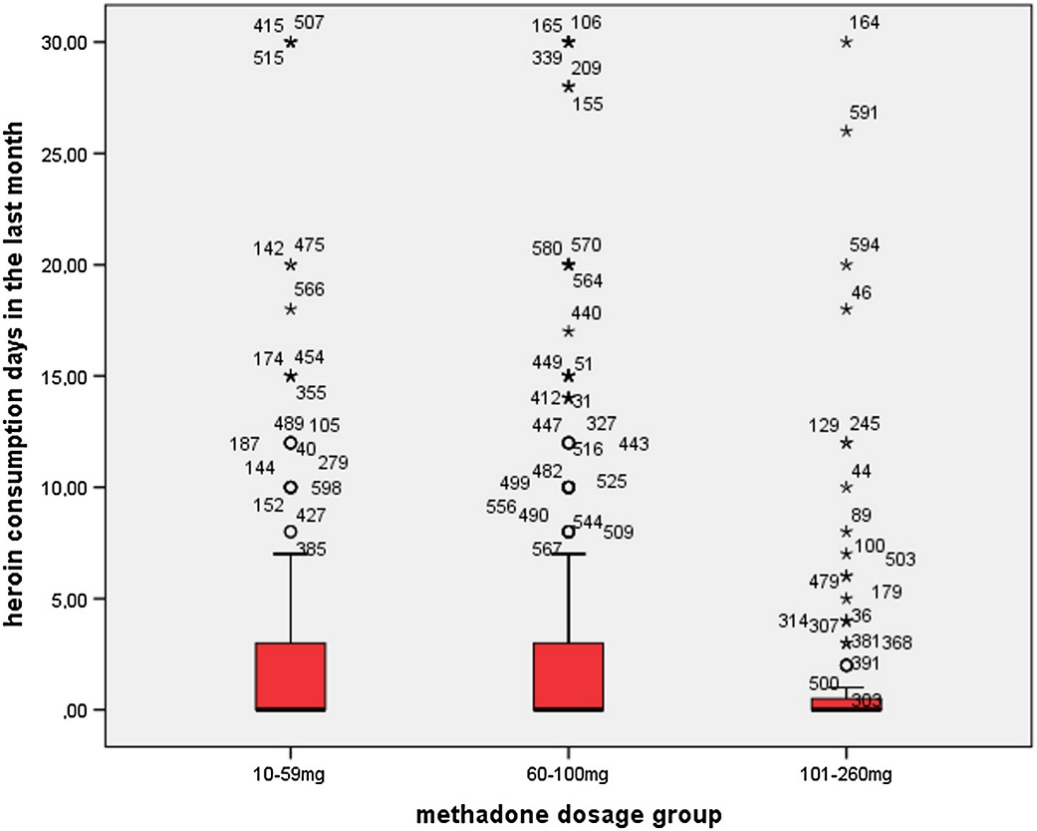
**Self-reported heroin use in 613 methadone-maintained patients during the 30 days prior to the interview differed significantly with the prescribed methadone dose (mg/ day) (Kruskal-Wallis Test; χ** ^**2**^ **= 13.326; df = 2; p < 0.001).** Patients in the high dose group reported significantly less heroin use compared to the low dose group (Mann–Whitney; Z = −2.723; p < 0.01) and the medium dose group (Mann–Whitney; Z = −3.645; p < 0.001).

Two thirds of the patients reported no concomitant heroin use at all in the 30 days (n = 390, 63.6%), and one third reported at least 1 heroin consumption day (n = 223, 36.4%). In the LDG, 37.0% (n = 74) reported heroin use, in the MDG 41.8% (n = 114) and in the HDG 25.0% (n = 35). Patients reported a mean of 2.8 ± 6.1 heroin consumption days in the 30 days prior interview, with significant differences between groups (p < 0.001) (see Figure 2). Patients in the HDG reported significantly fewer heroin consumption days compared to the LDG (p < 0.01) and the MDG (p < 0.001). Patients treated in specialized centers exhibited significantly higher proportions of concomitant heroin use than patients in office-based settings (39.8% versus 29.6%; χ^2^ = 6.138; df = 1; p < 0.05).

## Discussion

To our knowledge, this is the first register-based study addressing the association between prescribed methadone dose levels and concomitant cocaine use among a MMT population in Europe. The results show that self-reported concomitant cocaine consumption days were low in our study, with an average of 2.8 days in the past 30 days. Surprisingly, we found that patients in the LDG had significantly fewer cocaine consumption days than patients in the currently recommended dose range > 60 mg/d [17, 43]. Thus, we had to reject our hypothesis that higher methadone doses would result in lower concomitant cocaine use. This finding supports the already mentioned RCT of Kennedy et al. [41], which found that individualized dosages of methadone of up to 190 mg/day were not better than doses of 100 mg/day with respect to concomitant heroin and cocaine use. In contrast, in a recent CPS, Peles et al. [36] reported that high methadone doses (> 100 mg/day) may reduce cocaine use in patients addicted to both heroin and cocaine. However, these studies analyzed concomitant cocaine use with urine analyses and were not only based on self-report. Moreover, a recent Cochrane Review [17] with 11 RCTs and 10 CPS, concluded that methadone dosages ranging from 60 to 100 mg/day are more effective than lower dosages in retaining patients and reducing concomitant cocaine and heroin use. Nevertheless, additional studies are needed to examine the effects of higher methadone dosage levels (> 100 mg/d) on concomitant cocaine use.

The main finding in this study was that the most important factor predicting cocaine use in MMT was concomitant heroin use (OR 4.9), indicating that concomitant heroin and cocaine use is common in a subgroup of methadone patients. Furthermore, the odds for cocaine use were increased in patients with prescriptions of both medium (OR 2.3) and high (OR 2.1) methadone doses as compared to the LDG. One explanation could be drawn from the study results that patients in the LDG started cocaine and heroin use later in their lives and seem be more socially integrated, as the LDG is the group with significantly more employed patients compared to the MDG and HDG. Furthermore, the LDG probably has less poly-drug use and less concurrent medication for psychiatric comorbidities. Patients in specialized centers were more likely to have concomitant cocaine use (OR 1.9) than patients in office-based settings. This result corresponds to clinical practice, referring more instable patients to specialized centers. The same accounts for the significant result in less take-home medication days in patients with more concomitant cocaine use (Table 3).

Study results show that 67.3% of the patients had methadone doses prescribed in the recommended dose range according to Swiss treatment guidelines [13], and 32.6% had prescribed doses below 60 mg/day. As expected, we found that patients with prescribed methadone doses > 100 mg/day were associated with significantly fewer reported heroin consumption days compared to the LDG and MDG. Our findings are consistent with previous literature reviews [22] and observational studies [16, 21, 22, 25] and strongly support these. Overall, we found a low average number of concomitant heroin consumption days in our survey, with 2.8 consumption days in the 30 days prior interview. Of concern, however, is the fact that patients in the HDG had significantly more alcohol consumption days than patients in the LDG. This supports the findings of Gossop et al. [45] that patients maintained at high methadone doses include a higher proportion of heavy drinkers. The results in this register-based study also show that most patients enrolled in MMT in Basel City stay in treatment for long periods. Remarkably is the significantly longer length of stay in treatment in the HDG compared to the LDG.

### Limitations of the study

The results of this study were obtained through a retrospective analysis. Though this assures high external validity, prospective statements are not possible and causation between methadone dose and concomitant substance use cannot be drawn from a register-based study. Furthermore, this study does not provide information about patients staying for short periods in MMT (<1 year), nor about patients who did not attend the interviews; this might have led to underestimation of the amount of illicit cocaine and heroin use. Another limitation is that concomitant drug use in MMT is based on self-reported data and not verified by urine analyses. However, parallel drug use does not usually lead to exclusion from MMT. We are confident that self-reported drug use is quite similar to results from urine analyses. As Decker et al. observed, self-reported cocaine use and urine analyses demonstrate good concordance within treatment [46]. In a previous controlled trial of methadone-maintained patients, we found 58% heroin-negative urine samples, compared to 63.6% in this survey [12]. Furthermore, we had no information about the kind, frequency, amount and route of administration of concomitant drug use (e.g. injected heroin-cocaine-benzodiazepine-“cocktail”) that could be related to different methadone levels. Moreover, we had no information about patients’ psychiatric co-morbidities which could possibly influence concomitant cocaine and heroin use. Finally it must be emphasized that the data are nearly 10 years old. On the other hand, recent reports on cocaine use in Switzerland indicate stable cocaine prevalence in the last decade [10].

## Conclusions

Despite these limitations, the following conclusions can be drawn from the present study. Firstly, two thirds of methadone prescriptions to opioid-dependent patients were within the recommended range. Secondly, prescribed methadone doses > 100 mg/day (HDG) were not associated with significantly fewer cocaine consumption days but with fewer heroin consumption days. Thirdly, concomitant heroin use was the major risk factor for cocaine use in MMT. This means that methadone providers are required to raise awareness for this issue by prescribing appropriate methadone doses, in order to reduce concomitant heroin use directly and cocaine use indirectly.
